# Translation and validation of the Uterine Fibroid Symptom and Quality of Life (UFS-QOL) questionnaire for the Brazilian Portuguese language: Tradução e validação do questionário Uterine Fibroid Symptom and Quality of Life (UFS-QOL) para a língua portuguesa brasileira

**DOI:** 10.1590/1516-3180.2016.0223281016

**Published:** 2017-04-13

**Authors:** Luiz Gustavo Oliveira Brito, Daniela Alves Malzone-Lott, Mayra Fernanda Sandoval Fagundes, Pedro Sérgio Magnani, Mariana Alves Fernandes Arouca, Omero Benedicto Poli-Neto, Antônio Alberto Nogueira

**Affiliations:** I MD, PhD. Attending Physician, Department of Gynecology and Obstetrics, Faculdade de Medicina de Ribeirão Preto (FMRP), Universidade de São Paulo (USP), and School of Medicine, Universidade de Ribeirão Preto (UNAERP), Ribeirão Preto (SP), Brazil.; II MD. Physician, Department of Gynecology and Obstetrics, Faculdade de Medicina de Ribeirão Preto (FMRP), Universidade de São Paulo (USP), Ribeirão Preto (SP), Brazil.; III MD. Attending Physician, Department of Gynecology and Obstetrics, Faculdade de Medicina de Ribeirão Preto (FMRP), Universidade de São Paulo (USP), Ribeirão Preto (SP), Brazil.; IV MD, MSc. Attending Physician, Department of Gynecology and Obstetrics, Faculdade de Medicina de Ribeirão Preto (FMRP), Universidade de São Paulo (USP), Ribeirão Preto (SP), Brazil.; V MD, PhD. Associate Professor, Department of Gynecology and Obstetrics, Faculdade de Medicina de Ribeirão Preto (FMRP), Universidade de São Paulo (USP), Ribeirão Preto (SP), Brazil.

**Keywords:** Leiomyoma, Validation studies [publication type], Translations, Quality of life, Cross-sectional studies

## Abstract

**CONTEXT AND OBJECTIVE:**

Uterine fibroids (UF), also known as leiomyomas, are the most prevalent gynecological tumors. The Uterine Fibroid Symptoms and Quality of Life (UFS-QOL) is the only specific questionnaire that assesses symptom intensity and quality-of-life issues for women with symptomatic UF; however, it only exists in the English language. Thus, we aimed to translate and culturally validate the ­UFS-QOL questionnaire for the Brazilian Portuguese language.

**DESIGN AND SETTING:**

Cross-sectional study, Department of Gynecology and Obstetrics, FMRP-USP.

**METHODS:**

113 patients with UF (case group) and 55 patients without UF (control group) were interviewed using the UFS-QOL questionnaire after translation and cultural adaptation. The Short Form-36 questionnaire was used as a control. Demographic and psychometric variables were analyzed.

**RESULTS:**

Women with UF presented higher mean age, body mass index, weight, parity and comorbidities than the control group (P < 0.05). The most prevalent complaints were abnormal uterine bleeding (93.8%), pelvic pain (36.3%) and extrinsic compression (10.6%) and these presented adequate construct validity regarding UFS-QOL severity (P < 0.05). The UFS-QOL questionnaire presented good internal consistency regarding symptom severity and quality-of-life-related domains (intraclass correlation coefficient, ICC = 0.82/0.88). Structural validity presented correlation coefficients ranging from 0.59 to 0.91. Test-retest comparison did not show differences among the UFS-QOL subscales. After treatment, women with UF presented improvements on all subscales.

**CONCLUSION:**

The UFS-QOL questionnaire presented adequate translation to the Brazilian Portuguese language, with good internal consistency, discriminant validity, construct validity, structural validity and responsiveness, along with adequate test-retest results.

## RESUMO

**CONTEXTO E OBJETIVO:**

O leiomioma uterino (LU) é o tumor ginecológico mais comum. Existe apenas um questionário específico que avalia a intensidade de sintomas e qualidade de vida de mulheres com LU sintomático, o Uterine Fibroid Symptom and Quality of Life (UFS-QOL), porém somente na língua inglesa. Dessa forma, objetivamos traduzir e validar culturalmente o questionário UFS-QOL para a língua portuguesa brasileira.

**TIPO DE ESTUDO E LOCAL:**

Estudo transversal, Departamento de Ginecologia e Obstetrícia da FMRP-USP.

**MÉTODOS:**

113 pacientes portadoras de LU (grupo caso) e 55 pacientes-controle foram entrevistadas com o questionário UFS-QOL após tradução e adaptação cultural. O questionário Short Form-36 foi utilizado para controle. Variáveis demográficas e psicométricas foram analisadas.

**RESULTADOS:**

As mulheres com LU apresentaram maior média de idade, índice de massa corporal, peso, paridade e comorbidades do que o grupo controle (P < 0,05). As queixas mais prevalentes foram sangramento uterino anormal (93,8%), dor pélvica (36,3%) e compressão extrínseca (10,6%) e estas apresentaram adequada validade de constructo com a gravidade indicada pelo UFS-QOL (P < 0,05). O questionário UFS-QOL apresentou boa consistência interna com a gravidade dos sintomas e com os domínios relacionados a qualidade de vida (coeficiente de correlação intraclasse, CCI = 0,82/0,88). A validade estrutural mostrou coeficientes de correlação variando de 0,59 até 0,91. A comparação teste-reteste não mostrou diferença entre as subscalas do UFS-QOL. Depois do tratamento, as mulheres com LU apresentaram melhora em todas as subscalas.

**CONCLUSÃO:**

O questionário UFS-QOL apresentou adequada tradução para a língua portuguesa brasileira, com boa consistência interna, validade de constructo/discriminatória, estrutural e responsividade, assim como adequados resultados teste-reteste.

## INTRODUCTION

Uterine fibroids (UF), also known as leiomyomas, are the most common gynecological tumors and they originate from the smooth muscle cells of the uterine wall. Although 60 to 80% of women present these tumors,^1^ only 20 to 30% present symptoms, such as abnormal uterine bleeding and pelvic pain.^2^ In Brazil, there are no accurate epidemiological data for this disease; however, the number of hysterectomies performed in this country has stabilized,^3^ even though clinical management of UF is still a challenge.^4^

An observational study showed that UF had a negative impact on the patients’ quality of life.^5^ They presented social and professional limitations, with fear, disbelief and despondency regarding their symptoms, and these factors strengthened their will to undergo hysterectomy.^6^ Thus, the impact of UF symptoms on women’s health-related quality of life (HRQL) is a major indicator for treatment.

Questionnaires may be useful for measuring the impact of symptoms and the results from clinical and/or surgical interventions. Fourteen years ago, the only questionnaire directed towards UF, the Uterine Fibroid Symptom and Quality of Life (UFS-QOL) questionnaire, was published.^7^ The responsiveness of this questionnaire was subsequently assessed^8^ and it was validated in a non-randomized prospective study.^9^ Thus, many studies that have investigated UF have been using this questionnaire.

To our knowledge, from searching in the literature, there is no cultural translation/validation of the UFS-QOL questionnaire in the Brazilian Portuguese language. A simple translation is not valid for use, given that it is known that adapting questionnaires to the local language is a difficult task.^10^ Moreover, regional expressions should be incorporated and therefore there is a need to conduct a study to confirm the cultural validity of the questionnaire.

## OBJECTIVE

The authors aimed to translate and culturally validate the ­UFS-QOL questionnaire for the Brazilian Portuguese language.

## METHODS

### Type of study and patients

A cross-sectional study was conducted at the Gynecological Surgery Clinic of Hospital das Clínicas, Faculdade de Medicina de Ribeirão, Universidade de São Paulo (HCFMRP-USP), between April and September 2015. Women were recruited through the consultations that are provided in this setting and were treated by means of clinical or surgical management. The control group was constituted by women from other outpatient gynecological clinics at HCFMRP-USP who did not present abnormal uterine bleeding or pelvic pain as their main complaints.

The inclusion criteria were that the subjects should be women of fertile age presenting UF with complaints of abnormal uterine bleeding or pelvic pain or a sensation of external compression. We excluded pregnant women, those who were using anticoagulants or presented abnormal uterine bleeding of other secondary causes (endometrial or ovulatory), and those with cognitive impairments or who were illiterate.

All participants signed an informed consent statement and this study was approved by our Institutional Review Board (procedural number 531.584). This study was drawn up in accordance with the ethical guidelines of the 1975 Declaration of Helsinki.

### UFS-QOL questionnaire: translation and validation

The UFS-QOL questionnaire specifically assesses severity of symptoms (8 questions) and HRQL (29 questions) among women with UF. The HRQL scale comprises the following subscales: concern, activities, energy/mood, control, self-consciousness and sexual function.^7^ All answers have five options on a Likert scale. The higher the score on the severity subscale of the questionnaire is, the greater the severity of symptoms is; while the lower the scores on the HRQL subscales are, the poorer the quality of life is.

Written authorization was firstly obtained from the Society for Interventional Radiology and from Prof. James Spies to translate and validate the UFS-QOL questionnaire. Following this, the initial translation was performed by two notarized bilingual professors. This translation was evaluated by a committee composed of three gynecologists (LGOB, DAML and MAFA) and was pilot-tested on ten women who were attended in the outpatient setting. These patients were asked about any difficulties in comprehending the questionnaire. Some words were adapted by the interviewer to ease their understanding. The changes thus made were reviewed by the same medical committee and the final version was released for application. A back translation was performed by a native speaker of English who had knowledge of the Portuguese language. The final questionnaire was submitted to and received consent for publication from the Society for Interventional Radiology.^11^

There was no standardization regarding the mode of application of the questionnaire (oral interview or self-application). However, most of the questionnaires were self-administered by the patients, with the option of asking the interviewer for help if any queries arose while answering the questionnaire.

In addition, the Short Form-36 (SF-36) questionnaire was used as a control, to make comparisons with the UFS-QOL questionnaire and thus calculate its construct validity. The SF-36 questionnaire measures HRQL using eight domains: vitality, physical functioning, bodily pain, general health perceptions, physical role functioning, emotional role functioning, social role functioning and mental health.^12^ The individual items from each subscale are combined to form an overall score, which is then transformed into a score from 0 to 100. Higher scores indicate better HRQL.^13^

### Variables

The following demographic data were obtained: age, weight, height, body mass index (BMI), gravidity, parity and comorbidities. The following clinical symptoms were investigated: presence of abnormal uterine bleeding, pelvic pain and a sensation of external compression.

With regard to psychometric properties for measuring questionnaires, the variables analyzed were: internal consistency, construct validity, test-retest similarity and responsiveness. Internal consistency assesses the correlation between the items and is determined from the subscale and total scores. Higher values indicate higher correlation among several items on the scales, i.e. one or several items may be measuring the same concept. Values over 0.70 were considered adequate or acceptable.^14^ Construct validity was calculated by comparing the symptoms with the UFS-QOL score response and was considered adequate when different responses were perceived between the groups with and without symptoms. Structural validity was explored by means of principal-component analysis, with standardized coefficients comparing all questions from each quality-of-life subscale with the severity subscale, in order to identify the highest correlation coefficient. It is important to note that this psychometric analysis was previously done by Spies et al.,^7^ to individualize the subscales. A retest (n = 20) was performed 1-2 weeks after first application of the questionnaire. Responsiveness (n = 44) was assessed after three months of clinical and/or surgical treatment, to ascertain whether the UFS-QOL scores had changed after the first application of the questionnaire.

### Statistical analysis

The data were tabulated in Microsoft Excel (Microsoft Co., Redmond, WA, USA) and statistical analyses were performed by means of Intercooled Stata 13.0 (College Station, TX, USA). A significance level of 5% was stipulated in all two-sided tests. Normality analysis was performed using the Shapiro-Wilk test, and the continuous variables (age, body mass index and weight) were found to have parametric distribution. The chi-square test was used for binomial variables and Student’s t test for continuous variables.

No power calculation was performed, because of the extensive variability of formulae that have been suggested for generating the minimum number of subjects for a given study that involves translating and validating a standardized questionnaire.^10^

The internal consistency was calculated by means of Cronbach’s alpha (over 0.9, excellent; 0.7-0.9, acceptable to good; 0.6-0.7, questionable; 0.5-0.6, poor; and below 0.5, unacceptable) and by means of item correlation. The structural validity was calculated by means of structural equation modeling between severity and other quality-of-life subscales. Test-retest similarity and responsiveness were calculated using a paired t test, with extraction of the mean and standard deviation and the mean difference between variables.

Missing data were not treated using imputation methods. All patients answered at least 50% of the questions on both questionnaires.

## RESULTS

The study group was formed by 113 women and the control group by 55 women, thus totaling 168 patients recruited over the study period. We had 5 refusals to participate, and 12 women were excluded from the study (due to cognitive incapacity to understand the questionnaire) during the enrollment period.

The demographic data are described in Table 1. The women with UF presented higher mean age (42.6 ± 6.5 years; P < 0.005), weight (78.9 ± 16.7 kg), BMI (30.3 ± 6 kg/m²; P = 0.01) and parity (P < 0.005). The presence of comorbidities was higher among women with UF than in the control group, without any statistically significant difference (53.1% versus 38.2%; P = 0.069). The most common diseases in the study group were arterial hypertension and anxiety/depressive disorders. Among the clinical symptoms, abnormal uterine bleeding was the most prevalent (93.8%), followed by pelvic pain (36.3%) and external compression (10.6%).

**Table 1.** Demographic and clinical baseline characteristics

Variables	Control group (n = 55)	Study group (n = 113)	P-value
Age (mean ± standard deviation)	37.9 (8.2)	42.6 (6.5)	< 0.005
Gravidity (median/range)	1 (0-6)	2 (0-6)	< 0.005
Parity (median/range)	1(0-5)	2(0-6)	< 0.005
Educational level (n, %)			
0-6 years	15 (27.3)	44 (38.9)	< 0.005
7-12 years	21 (38.2)	63 (55.9)	
> 12 years	18 (32.7)	3 (2.6)	
Not informed	1 (1.8)	3 (2.6)	
Body mass index (mean ± standard deviation)	27.7 (5.8) (n = 54)	30.3 (6.0) (n = 110)	0.010
Comorbidities (n, %)	21 (38.18)	60 (53.1)	0.069
Symptoms			
Abnormal uterine bleeding	1 (1.8)	106 (93.8)	< 0.005
Pelvic pain	3 (5.5)	41 (36.3)	< 0.005
External compression	1 (1.8)	12 (10.6)	< 0.005

Table 2 shows the analysis of the UFS-QOL and SF-36 scores in both groups. All UFS-QOL subscales showed statistically significant differences between women with UF and controls (P < 0.05). The women with UF presented higher severity scores and lower scores on the subscales that measured quality of life, compared with the controls. The self-consciousness and sexual function subscales presented greater reductions in mean difference scores between the women with UF and the others. Regarding the SF-36 domains, women with UF presented lower quality of life (P < 0.05) except for the vitality domain (P = 0.07). Thus, discriminant validity could be demonstrated.

**Table 2.** Discriminant validity of the Uterine Fibroid Symptom and Quality of Life (UFS-QOL) and Short-Form-36 (SF-36) questionnaires among women with uterine fibroids and control patients

Questionnaires	Mean (SD)		P-value
	Control group	Study group	
	(n = 55)	(n = 113)	
UFS-QOL			
Symptom severity	15.7 (17.6)	59.4 (19.1)	< 0.001
Concern	95.2 (10.9)	33.6 (28.7)	< 0.001
Activities	96.5 (10.6)	53.8 (26.0)	< 0.001
Energy/Mood	92.9 (18.6)	47.3 (28.5)	< 0.001
Control	94.5 (16.2)	51.3 (27.3)	< 0.001
Self-consciousness	94.7 (16.2)	49.3 (25.7)	< 0.001
Sexual function	90.7 (23.8)	43.9 (36.6)	< 0.001
Total Score	361.7 (73.7)	115.7 (116.9)	< 0.001
SF-36			
Physical functioning	74.9 (32.3)	59.2 (29.6)	0.0021
Physical role functioning	73.2 (39.0)	38.3 (44.9)	< 0.001
Bodily pain	62.5 (26.2)	52.0 (28.4)	0.023
General health perceptions	58.6 (17.9)	50.5 (20.2)	0.013
Vitality	56.9 (21.7)	49.6 (25.8)	0.072
Social role functioning	74.8 (30.7)	59.9 (32.0)	0.003
Emotional role functioning	72.7 (40.1)	44.8 (44.5)	< 0.001
Mental health	60.7 (21.5)	50.8 (26.5)	0.017

The internal consistency calculation on the UFS-QOL questionnaire is shown in Table 3. Values over 0.75 were found for all its subscales and for the HRQL score in the control and study groups, thus showing that the concordance was adequate. Item correlation presented moderate concordance regarding symptom severity. Similarly, in the study group, moderate concordance was seen regarding symptom severity, self-consciousness and sexual function. The construct validity showed statistical significance between the presence of symptoms and higher mean UFS-QOL severity score (Table 4). With regard to responsiveness, all UFS-QOL subscales presented improvement in quality of life and reduction in the severity of symptoms after treatment (Table 5). The analysis on standardized coefficients for quality-of-life subscales, with severity as covariance, showed values ranging from 0.59 to 0.91, as seen in Table 6. The covariance analysis with severity showed highest correlations with the following subscales: concern (0.91), control (0.80) and self-consciousness (0.81). Finally, no differences were noted in the test-retest comparison of the UFS-QOL version for the Brazilian Portuguese language (Table 7). The final version is presented with this paper (Annex**)**.

**Table 3.** Internal consistency (ICC) and item correlation among women with uterine fibroids and control group

UFS-QOL	Control group (n = 55)		Study group (n = 113)	
	Item correlation	Cronbach’s alpha	Item correlation	Cronbach’s alpha
Symptom severity	0.59	0.84	0.65	0.82
Concern	0.84	0.84	0.80	0.80
Activities	0.89	0.83	0.81	0.81
Energy/mood	0.96	0.81	0.86	0.80
Control	0.92	0.82	0.86	0.80
Self-consciousness	0.90	0.82	0.66	0.82
Sexual function	0.90	0.80	0.72	0.81
HRQL score	1.00	0.91	1.00	0.88

UFS-QOL = Uterine Fibroid Symptom and Quality of Life; HRQL = health-related quality of life.

**Table 4.** Construct validity between uterine fibroid symptoms and the Uterine Fibroid Symptom and Quality of Life (UFS-QOL) questionnaire

	Study group	Control group	Mean difference	P-value
	Mean ± standard deviation			
Abnormal uterine bleeding	59.57 ± 19.92	19.67 ± 20.10	-39.9	< 0.001
Pelvic pain	60.51 ± 20.15	39.61 ± 28.02	-20.89	< 0.001
External compression	56.00 ± 23.74	44.17 ± 27.88	-11.83	0.014

**Table 5.** Responsiveness of women with uterine fibroids after treatment (n = 44)

UFS-QOL	Before treatment	After treatment	Mean difference	P-value
	Mean/standard deviation			
Symptom severity	54.97 (14.57)	15.05 (17.91)	-39.91	< 0.005
Concern	32.84 (24.97)	86.59 (24.97)	+53.75	< 0.005
Activities	57.95 (24.96)	91.72 (13.81)	+33.76	< 0.005
Energy/mood	50.08 (28.37)	83.76 (22.69)	+33.69	< 0.005
Control	54.32 (26.86)	87.61 (20.50)	+33.29	< 0.005
Self-consciousness	50.95 (22.68)	75.19 (27.70)	+24.24	< 0.005
Sexual function	43.18 (36.21)	65.91 (39.19)	+22.72	< 0.005
HRQL score	124.42 (108.26)	298.09 (110.96)	+173.68	< 0.005

UFS-QOL = Uterine Fibroid Symptom and Quality of Life; HRQL = health-related quality of life.

**Table 6.** Structural validity of all quality-of-life subscales compared with the severity scale of the UFS-QOL questionnaire by means of structural equation modeling

UFS-QOL questionnaire (model showing interaction of quality-of-life subscales with the severity subscale)	Standardized coefficient (standard error)	Variance
Concerns (last 3 months)		
Anxiety about unpredictable onset/duration of periods (Q9)	0.78 (0.031)	0.38
Soiling underclothes (Q15)	0.91 (0.015)	0.16
Soiling bed linen (Q22)	0.88 (0.018)	0.21
Feeling inconvenienced about carrying extra pads (Q28)	0.67 (0.044)	0.54
Soiling outer clothes (Q32)	0.91 (0.015)	0.15
Activities (last 3 months)		
Anxiety about traveling (Q10)	0.62 (0.050)	0.60
Interfered with physical activities (Q11)	0.77 (0.036)	0.39
Decreased amount of time on exercise (Q13)	0.73 (0.041)	0.45
Felt that activity was difficult to carry out (Q19)	0.78 (0.034)	0.38
Interfered with social activities (Q20)	0.78 (0.034)	0.38
Plan activities more carefully (Q27)	0.82 (0.028)	0.31
Caused embarrassment (Q29)	0.78 (0.034)	0.38
Energy (last 3 months)		
Felt tired (Q12)	0.81 (0.029)	0.34
Felt drowsy or sleepy during day (Q17)	0.72 (0.039)	0.47
Felt sad, discouraged or hopeless (Q23)	0.88 (0.020)	0.21
Felt downhearted and blue (Q24)	0.88 (0.019)	0.21
Felt wiped out (Q25)	0.86 (0.022)	0.25
Felt irritable (Q31)	0.83 (0.025)	0.29
Felt weak as energy drained from her body (Q35)	0.87 (0.020)	0.24
Control (last 3 months)		
Felt that she was not in control of her life (Q14)	0.79 (0.033)	0.37
Felt less productive (Q16)	0.78 (0.034)	0.38
Concerned about her health (Q26)	0.84 (0.027)	0.29
Felt uncertain about her future (Q30)	0.77 (0.035)	0.39
Felt that she was not in control of her health (Q34)	0.84 (0.028)	0.29
Self-consciousness (last 3 months)		
Felt self-conscious of weight gain (Q18)	0.71 (0.048)	0.49
Felt conscious about the size and appearance of her stomach (Q21)	0.80 (0.041)	0.35
Affected the size of clothing she wore during her periods (Q33)	0.59 (0.058)	0.64
Sexual function (last 3 months)		
Diminished sexual desire (Q36)	0.90 (0.031)	0.17
Caused patient to avoid sexual intercourse (Q37)	0.90 (0.032)	0.18
Covariance severity		
Concern	0.91 (0.018)	-----------
Activity	0.79 (0.036)	-----------
Energy	0.78 (0.034)	-----------
Control	0.80 (0.035)	-----------
Self-consciousness	0.81 (0.043)	
Sexual function	0.65 (0.050)	-----------

**Table 7.** Test-retest similarity of the UFS-QOL questionnaire in the study group (n = 20)

UFS-QOL	Test	Retest	Mean difference	P-value
	Mean (standard deviation)			
Symptom severity	57.19 (19.35)	52.03 (32.81)	-5.16	0.453
Concern	39.5 (33.34)	48.25 (35.37)	+8.75	0.088
Activities	54.8 (27.79)	61.79 (30.35)	+6.96	0.270
Energy/mood	54.64 (32.36)	54.11 (33.83)	-0.53	0.929
Control	53.5 (27.29)	60 (32.16)	+6.5	0.223
Self-consciousness	50.83 (31.75)	53.33 (32.82)	+2.5	0.746
Sexual function	46.25 (35.14)	45 (39.82)	-1.25	0.892
HRQL score	133.23 (133.27)	152.99 (158.69)	+19.77	0.452

UFS-QOL = Uterine Fibroid Symptom and Quality of Life; HRQL = health-related quality of life.

## DISCUSSION

This study showed that in the UFS-QOL questionnaire, the symptoms of women with UF strongly correlated with lower HRQL subscales and higher severity scores, and thus that the women’s symptoms had an impact on their quality of life. No differences with regard to the ICC were seen on the UFS-QOL subscales among the women with UF, although item correlation was moderate among the symptoms of severity, self-consciousness and sexual function. These data are similar to what was found in the validation studies conducted by Spies et al. and Coyen et al.^7^^,^^9^ Moreover, women with UF presented worse quality of life when analyzed by means of the SF-36 questionnaire, except for the vitality domain. This may indicate that the UFS-QOL questionnaire is fulfilling its task of targeting women with UF.

With regard to the structural validity of the UFS-QOL questionnaire, we prepared structural equation modeling to compare the severity subscale with the other subscales, with the aim of determining which of them presented the highest correlation coefficient. We did not analyze all possibilities because this had already been done by Spies et al.^7^ when they published the UFS-QOL questionnaire in the English language. Structural validity is usually determined when a questionnaire is initially launched, to prove that the questions are correctly divided between the best-fit subscales.

Our test-retest reliability determination showed through the paired t test that there was no systematic difference at the second visit, after an interval ranging from 1 to 2 weeks. This is similar to what Spies et al.^7^ found with 27 patients and they did not consider the sample size to be underpowered for detecting differences in baseline characteristics. Our total sample size was larger than that of the first publication of this questionnaire.^7^

Furthermore, the UFS-QOL questionnaire for the Brazilian Portuguese language also demonstrated responsiveness to change among women treated with medications or surgery, with mean change scores greater than 20 points for all subscales. Although only 40 patients were included, these data are supported by those of Harding et al.,^8^ who analyzed 102 patients with UF who were treated with thermal ablation by means of MRI-guided focused ultrasound (MRgFUS); and by data from larger studies such as the Fibroid Registry for Outcomes Data (FIBROID), a database in which more than 2,000 women undergoing uterine embolization for leiomyomata were studied.^15^ Recently, the questionnaire was slightly modified for women who have undergone hysterectomy.^16^ Some non-English-speaking countries have used this questionnaire for studies on women with UF without validating it, such as a study conducted in Korea.^17^

This study presented some limitations: we did not calculate the criterion validity, which would consist of assessment of the UFS-QOL questionnaire using a clinical tool that is considered to be the gold standard. In this case, we would have had to compare it with the alkaline hematin technique for quantifying blood loss, or with a pictorial blood assessment chart (PBAC). On the other hand, although the control and study groups were different with regard to age and comorbidities, we do not believe that these variables caused a great impact on our results, just as stated by Spies et al., probably because women with UF present epidemiological features that would be difficult to create in a similar control group.^7^

We believe that we met our goal of confirming that the UFS-QOL questionnaire could be translated without language impairment. Thus, we confirmed that the Brazilian Portuguese version is adequate for identifying women with UF, in the same way that it does this in English-speaking countries. Our expectation is that the translation and validation of this questionnaire will be very useful as a tool for measuring the impact of symptoms and the results from interventions relating to UF in Brazil and will strengthen future data produced in our setting.

## CONCLUSION

The Brazilian Portuguese version of the UFS-QOL questionnaire is a valid and reliable instrument for assessing the HRQL of women with UF. It demonstrated good internal consistency, discriminant validity, construct validity, structural validity, test-retest similarity and responsiveness.
